# ReverseDock: a web server for blind docking of a single ligand to multiple protein targets using AutoDock Vina 

**DOI:** 10.3389/fmolb.2023.1243970

**Published:** 2023-10-10

**Authors:** Fabian Krause, Karsten Voigt, Barbara Di Ventura, Mehmet Ali Öztürk

**Affiliations:** ^1^ Signaling Research Centres BIOSS and CIBSS, University of Freiburg, Freiburg, Germany; ^2^ Institute of Biology II, University of Freiburg, Freiburg, Germany; ^3^ Institute of Biology III, University of Freiburg, Freiburg, Germany

**Keywords:** AutoDock Vina, web server, drug design, off-target analysis, blind docking

## Abstract

Several platforms exist to perform molecular docking to computationally predict binders to a specific protein target from a library of ligands. The reverse, that is, docking a single ligand to various protein targets, can currently be done by very few web servers, which limits the search to a small set of pre-selected human proteins. However, the possibility to *in silico* predict which targets a compound identified in a high-throughput drug screen bind would help optimize and reduce the costs of the experimental workflow needed to reveal the molecular mechanism of action of a ligand. Here, we present ReverseDock, a blind docking web server based on AutoDock Vina specifically designed to allow users with no computational expertise to dock a ligand to 100 protein structures of their choice. ReverseDock increases the number and type of proteins a ligand can be docked to, making the task of *in silico* docking of a ligand to entire families of proteins straightforward. We envision ReverseDock will support researchers by providing the possibility to apply inverse docking computations using web browser. ReverseDock is available at: https://reversedock.biologie.uni-freiburg.de/

## Introduction

Drug design efforts have benefited from the recent expansion of the small-molecule chemical space (Polishchuk et al., 2013). However, ligands chosen for a specific target protein may inadvertently inhibit other proteins within a particular pathway (Xie et al., 2011), or a ligand may bind multiple proteins from distinct pathways (Zhou et al., 2019). Several computational approaches are being developed to identify the target protein of a given ligand (Moumbock et al., 2019; Galati et al., 2021). Among these strategies, in cases where both the ligand and protein are novel, the utility of reverse docking protocols becomes evident, as machine learning and similarity-based screenings require previously known structures of protein–ligand pairs. Despite the increasing number of crystal structures, a significant proportion of potential protein–ligand complex structures remain uncharacterized. Consequently, reverse docking protocols emerge as a promising tool to bridge this gap.

While a few web servers exist for docking a ligand to multiple proteins, such as Acid (Wang et al., 2019), TarFisDock (Li et al., 2006) (offline as of 17.11.2022), and idTarget (Wang et al., 2012) (offline as of 17.11.2022), all of these servers restrict the analysis to pre-selected human drug target proteins (809, 698, and 2091, respectively). This limitation poses a challenge for users wishing to explore a list of proteins based on their interests. Conversely, Webina (Kochnev et al., 2020) and SeamDock (Murail et al., 2021) web servers permit users to submit their preferred ligands and proteins; however, this is limited to a single ligand–protein pair. As a result, a gap currently exists in the availability of a docking service capable of docking a given ligand to multiple user-submitted proteins. AutoDock Vina is one of the most commonly used open-source molecular docking software designed for the accurate prediction of protein–ligand interactions (Trott and Olson, 2010). It employs a hybrid search algorithm by combining genetic algorithms with a scoring function based on empirical binding affinity data (Trott and Olson, 2010). The hybrid search algorithm allows AutoDock Vina to efficiently explore the conformational space of ligands within a protein’s binding site, identifying energetically favorable binding modes and estimating binding affinities (Eberhardt et al., 2021). AutoDock Vina’s computational efficiency, combined with its ability to predict binding interactions with high accuracy, has made it an indispensable tool in virtual screening, lead optimization, and structure-based drug design. Furthermore, AutoDock Vina has two orders of magnitude speed and better docking pose accuracy compared to AutoDock 4, making it an ideal tool for high-throughput virtual screening applications (Chang et al., 2010; Nguyen et al., 2020). To bring the full power of docking to the experimental community, we developed ReverseDock, an AutoDock Vina-based, easy-to-use blind docking web server allowing users to freely select multiple protein targets for docking their ligand of interest. Furthermore, we demonstrate that among randomly selected proteins, ReverseDock is able to successfully capture the ranking and docking poses of ligands.

## Methods

### Preparation of docking files

The user can upload a ligand (.mol2) along with their preferred Protein Data Bank (PDB) structures (up to 100 structures, with less than 1,000 amino acids to minimize the risk of job failures due to an extensive search space). PDB files are first processed to remove nucleic acids, and then PDBFixer software (https://github.com/caiyingchun/pdbfixer) is applied to add missing amino acids, replace non-standard amino acids, remove heterogen atoms like water, and add missing heavy atoms. Finally, protonation of the ligand at pH 7 is achieved using Open Babel (O’Boyle et al., 2011), while proteins are protonated using the PROPKA method (Olsson et al., 2011) through pdb2pqr (Dolinsky et al., 2007).

### Docking of the submitted ligand by AutoDock Vina

For both input and output purposes, AutoDock Vina (Trott and Olson, 2010) employs the PDBQT (PDB with charges and atom types) molecular structure file format, which includes information about the ligand’s structure, atom types, charges, and torsional degrees of freedom. PDBQT files also contain ROOT, ENDROOT, BRANCH, and ENDBRANCH keywords that are recognized by AutoDock Vina, which establish the torsion tree of the submitted ligand .mol2 file. As such, various rotamers of the submitted ligand can be generated during docking simulations. In ReverseDock, AutoDock Vina required PDBQT files are generated by using the MGLTools software (Morris et al., 2009). The search space for docking is defined as a box with an edge 30 Å larger than that of the target protein in order to avoid steric restrictions on the ligand’s possible binding positions to the target. As recommended by previous studies for the converged docking poses with AutoDock Vina (Agarwal and Smith, 2023), a fixed exhaustiveness score of 64 has been selected for all docking calculations. Exhaustiveness determines the number of iterations and poses that AutoDock Vina will explore during the docking process (Agarwal and Smith, 2023). A higher exhaustiveness value indicates that the software will explore a larger number of possible binding orientations and conformations for the ligand within the binding site. Despite its computational cost, this is beneficial to increase the likelihood of finding the optimal binding pose and improve the accuracy of the predicted binding affinity between the ligand and the protein. In ReverseDock, flexible ligand docking is applied for the docking of each ligand to the submitted proteins by AutoDock Vina (Trott and Olson, 2010).

### Presentation of results

The results are displayed in a table, which are sorted by the calculated binding energy in kcal/mol. Each individual top docking ligand pose can be downloaded in PDBQT format. Additionally, each protein–ligand complex can be viewed in a 3D mode for quick analysis with the option to take a snapshot image of the docking pose.

### Web server development

ReverseDock employs a microservice architecture that enables flexible scaling. For instance, docking simulations can be distributed across multiple interconnected computers. Services are tasked with docking using AutoDock Vina (Eberhardt et al., 2021), and preparing receptors and ligands for docking using MGLTools (Olsson et al., 2011). The entire process concludes with e-mail dispatch. All services are scripted in Python. Communication between services occurs via the AMQP protocol. Submissions are queued in a manner that optimizes resource usage; a submission can initiate computation without waiting for a prior submission to complete, provided resources are available. The outward-facing API is coded in .NET 6.0, adhering to the Controller–Service–Repository pattern. For persistent data storage, MongoDB is employed, and Redis functions as temporary caching. The front end is crafted using TypeScript, React, and Blueprint, with NGLViewer (Morris et al., 2009) deployed for 3D molecular representation.

Upon submitting a .mol2 file, followed by up to 100 .pdb files or UniProt IDs for retrieving AlphaFold structures, should the user choose to proceed, tasks are disseminated through AMQP for relevant services to consume. Computation commences once resources are at hand, with interim results exhibited on the webpage. Upon processing all targets, an email notification is sent if the user has supplied one during submission.

## Demonstration cases

To evaluate the ranking and docking pose accuracy of ReverseDock, we created a list of random protein structures consisting of the following PDB IDs: 1udt, 2oyu, 3g6z, 3pbl, 2nnq, 3kba, 1uyg, 2uz3, 2hzi, 4ldo, 2i0e, 1sqt, 3m2w, 2oj9, 3erd, 3f9m, 1w7x, 2bgs, 2azr, and 2ica. Next, we extracted ligand .mol2 files from these PDB files and applied cross-docking calculations on ReverseDock. The results indicate that ReverseDock is able to identify the correct binding site in 75% (16/20) of the cases and can rank the corresponding protein–ligand complex in the top three positions in 50% (10/20) of the cases (Table 1), demonstrating that our tool can be used for target enrichment purposes of a given ligand. In addition to successful ranking, the best docking positions are also found to have an RMSD smaller than 3Å compared to the crystal structure in 55% (11/20) of the cases. A detailed inspection of incorrect binding site predictions indicates that buried, relatively large, or small ligands are not correctly identified.

**TABLE 1:** Cross-docking results are obtained by docking each ligand from the indicated PDB structure to all other PDB structures using ReverseDock. The ranking of the target PDB and the ligand RMSD of the first docking pose compared to the crystal structure is provided.

PDB structure	Docking ranking	Ligand RMSD of the first docking pose (Å)
1udt	1	8.4
2oyu	8	NA—wrong site
3g6z	2	0.6
3pbl	6	7.1
2nnq	1	0
3kba	1	0.4
1uyg	1	2.9
2uz3	1	1.5
2hzi	14	11.5
4ldo	5	NA—wrong site
2i0e	5	6.1
1sqt	5	1.4
3m2w	1	0.3
2oj9	3	0.8
3erd	2	0.4
3f9m	8	NA—wrong site
1w7x	6	1.8
2bgs	17	NA—wrong site
2azr	2	2.1
2ica	17	7.2

## Discussion

As demonstrated by the cross-docking results presented previously, through the utilization of AutoDock Vina with a pre-defined box size 30 Å larger than the target protein’s box size and an exhaustiveness score of 64 (Agarwal and Smith, 2023), ReverseDock can rank docking energies and reproduce the docking pose of the previously identified protein–ligand complex structures.

To ensure the quality and reliability of ReverseDock outputs, it is essential to address potential caveats and pitfalls that could impact the accuracy of the results. The scoring function utilized by Autodock Vina to estimate binding energies comes with limitations. Users should exercise caution when interpreting binding energies, as they may not always precisely reflect experimental results. Furthermore, the accuracy of docking simulations is dependent on the precision of the generated conformations and the extent of conformational space sampling.

Various strategies can be applied to evaluate the ReverseDock results. Visual inspection via molecular visualization software would be helpful in assessing the alignment of predicted binding poses with previously identified protein–ligand complexes. Comparing predicted binding sites with references from experimental structures or literature could aid in assessing the consistency and accuracy of predictions. Employing consensus scoring by using alternative docking tools can enhance confidence, particularly when multiple tools validate a specific binding mode. While binding energies may not be directly comparable to experimental data, comparing relative energies within a ligand set offers insights into relative affinities. Validation against existing data on analogous protein–ligand systems would also be helpful in assessing the quality of the predictions.

Ultimately, the integration of computational predictions with experimental validations, such as binding assays and advanced structure determination techniques such as X-ray crystallography or NMR spectroscopy, is recommended to establish the reliability and relevance of docking results.

We believe that our tool would be valuable for experimental researchers aiming to conduct reverse docking protocols to identify the target of a given ligand.

## Data Availability

The complete source code of ReverseDock as well as the input data used for demonstration cases can be accessed at: https://github.com/orgs/ReverseDock/repositories.
